# Isolated Central Nervous System Vasculitis Associated with Antiribonuclear Protein Antibody

**DOI:** 10.1155/2011/495201

**Published:** 2011-07-14

**Authors:** Amer M. Awad, Mathew Stevenson

**Affiliations:** ^1^Baton Rouge Neurology Associates, Baton Rouge General Medical Center, Baton Rouge, LA 70806, USA; ^2^Department of Neurology, University of Texas Southwestern Medical Center, Dallas, TX 75390-7208, USA

## Abstract

We describe the case of a young woman who was referred to a tertiary care center with unexplained subacute progressive encephalopathy preceded by long-standing severe headaches. Her extensive workup was remarkable for abnormal intracranial angiography suggestive of small- and medium-vessel vasculitis, persistently elevated protein in the cerebrospinal fluid and persistently high titers of antiribonuclear protein antibody. The patient showed a modest response to intravenous high-dose steroids. We propose that the patient's neurologic disease is secondary to immune-mediated central nervous system vasculitis, possibly as an initial manifestation of mixed connective tissue disease.

## 1. Introduction

Neurologic manifestations of rheumatic disease have been appreciated for many years [1]. Classically, mixed connective tissue disease (MCTD) has not been associated with severe central nervous system (CNS) disease [2]. However neurologic manifestations are seen in 10%–20% of patients with MCTD including headache, seizure, encephalopathy, aseptic meningitis, and neuropathy [1, 3]. MCTD is defined by the clinical syndrome and the presence of high titers of antibodies to an Rnase-sensitive ribonuclear protein complex, U1 small nuclear RNP (anti-RNP) [3]. The presence of these antibodies is highly sensitive (>98%) for MCTD with a reported specificity of 60% [4]. High titers give a higher specificity which may go as high as 92% [4–6]. Matsui and coworkers reported a case of a 69-year-old woman with a diagnosis of MCTD that initially presented with neurological features [1]. We present the case of a woman with severe headaches and encephalopathy in the presence of high titers of anti-RNP antibodies and imaging suggestive of small-to-medium-vessel vasculitis in the CNS. We emphasize the need not to overlook MCTD as a potential diagnosis in the presence of severe CNS pathology and to recognize neurologic presentation as the initial presentation of MCTD and other connective tissue diseases.

## 2. Case Report

The patient is a 47-year-old right-handed Caucasian woman with a history of left frontal meningioma and intractable headaches for more than 15 years. The headaches are described as dull holocephalic headaches with no light or sound sensitivity. Treatment of the left frontal meningioma by surgical resection and gamma-knife radiation for recurrence did not provide sustained relief of the patient's headache. Likewise, treatment for possible hydrocephalus with placement of ventriculoperitoneal shunt with multiple revisions did not provide any relief, and the shunt was removed by age 42. Since the age of 46, the patient was reported to have progressive cognitive decline and frequent falls. She was admitted to a local hospital and diagnosed with myasthenia gravis (MG). She was started on pyridostigmine, and after a brief rehabilitation, she was discharged home. On followup with a neurologist, the diagnosis of MG was challenged and excluded based on the clinical picture, serological testing (negative antiacetyl choline receptor antibody), and neurophysiological testing (negative repetitive nerve stimulation). Hence, pyridostigmine was discontinued, and the patient was started on valproic acid for headache control. Three months prior to admission to our tertiary center, the patient continued to experience a rapidly progressive cognitive decline. By the time the patient presented to our institution, she was bedridden and almost nonverbal.

**Figure 1:**
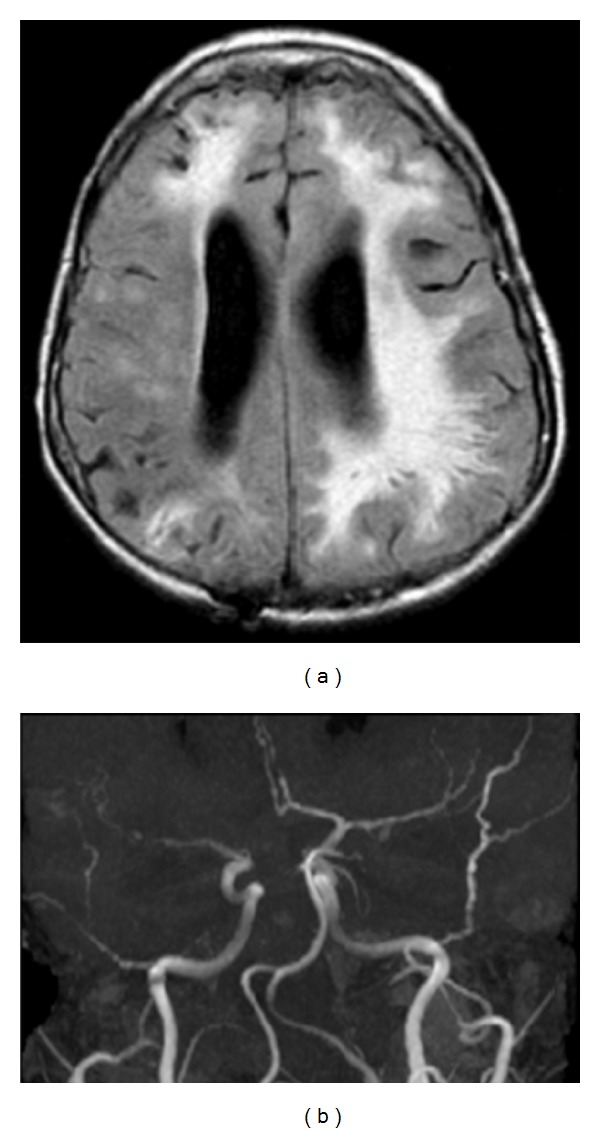
(a) Three mm axial fluid attenuated inversion recovery (FLAIR) brain magnetic resonance images (MRIs) demonstrating extensive subcortical white matter hyperintensity. (b) Magnetic resonance angiogram (MRA) demonstrating beading of small and medium intracranial vessels.

 On admission, the patient was noted to be tachycardic but afebrile. She was awake but unable to answer questions or follow any commands. Her spontaneous speech consisted only of repeated short phrases. She was noted to move her left side spontaneously but not the right side. Plantar reflexes were flexor on the left and extensor on the right (Babinski sign on the right). Laboratory investigations were remarkable for positive antiribonuclear protein (RNP) antibody at high titer in the serum and high protein levels (378 mg/dL) with normal cell count in the cerebrospinal fluid (CSF). Extensive infectious disease workup including viral, bacterial, and fungal studies were reproducibly negative. Magnetic resonance (MRI) of the brain, arteriography (MRA), and venography (MRV) were performed. The most striking finding was “beading appearance” of small- and medium-sized intracranial vessels bilaterally with multiple regions of white matter hyperintensities (Figures 1(a) and 1(b)).

 The patient was started on a course of high dose intravenous methylprednisolone (IVMP) (1 gram IV daily for 5 days). Her neurologic status showed modest improvement. She was able to follow one-step commands and respond to direct questions with simple sentences though her spontaneous speech continued to consist only of repeated phrases and moaning. Repeated MRA showed progression of the disease with severe attenuation of intracranial blood vessels. Repeated lumbar puncture showed an opening pressure of 13 cm H_2_O with high protein (103 mg/dL) with normal glucose and cell count. 

 A leptomeningeal biopsy was done and showed mild chronic perivascular inflammation and fibrosis with reactive astrocytosis. The anti-RNP antibody test was repeated and showed persistently high titers. The patient's husband declined to more aggressive immunosuppression like cyclophosphamide. The patient was discharged to a nursing home and died within six months of discharge.

## 3. Discussion

Sharp first described MCTD in 1972 [7], and the diagnostic criteria (and even the very existence of the disease) are debated to the present day [8]. Three widely used sets of diagnostic criteria include those by Sharp [9], those by Alarcon-Segovia and Villarreal [5] and sharp et al. [6], and the so-called “Japanese” criteria [10]. The common thread among all three criteria is the prominent role of anti-RNP in the diagnosis. Only Sharp's criteria do not implicate anti-RNP positivity as necessary for the diagnosis of MCTD [5, 6, 9, 10].

 Anti-RNP antibodies appear to be pathogenic [8, 11], and their disappearance is associated with periods of remission in MCTD [12]. The production of anti-RNP antibodies may be induced by molecular mimicry, possibly involving influenza B matrix protein, retroviral p30gag antigen [13], cytomegalovirus, and Epstein-Barr virus [11]. Subclasses of anti-RNP antibodies may be associated with distinct clinical scenarios [14]. In addition, some HLA subtypes are associated with specific patterns of tissue injury in the presence of anti-RNP antibodies [15].

 Vasculitis of the central nervous system typically presents with headache, mental status changes, and elevated protein in the CSF [16]. Left untreated patients can progress to develop focal signs, seizures, aphasia, hemiparesis, and coma [17]. 

 Intracranial vasculitis can be a primary disorder (variably known as primary CNS vasculitis (PCNSV), primary angiitis of the CNS (PACNS), and granulomatous angiitis of the CNS (GACNS)), or due to secondary causes. Secondary causes of CNS vasculitis include infectious (hepatitis B and C, herpes viruses, HIV, aspergillosis, coccidiomycosis, candidiasis, mucormycosis, rickettsia, mycobacterium, bacterial meningitis, and borrelia and treponemal infection), connective tissue disorders, other systemic vasculitides, and toxic (specifically amphetamine-induced) and paraneoplastic vasculitis [17]. The diagnosis of PACNS is based on the history of an acquired neurologic deficit, presence of histologic or angiographic evidence of vasculitis, and exclusion of other causes including infection [18]. The gold standard for diagnosis of CNS vasculitis is brain and leptomeningeal biopsy; however, the sensitivity has been reported to be only around 50% and negative predictive value of only around 70% [19]. Therapy consists of treating any underlying secondary causes and in the absence of these causes treating with high-dose steroids with or without subsequent cyclophosphamide [17].

 Our patient presented with chronic, severe headaches and progressive subacute encephalopathy with development of focal deficits. Extensive infectious, inflammatory, and paraneoplastic workup revealed only consistently elevated CSF protein and anti-RNP at high titer and evidence of medium-vessel vasculitis on MRA. Paraneoplastic panel was negative. In addition, lactate and pyruvate in the serum and the CSF were normal which potentially excluding mitochondrial encephalopathy. 

 As mentioned earlier, sensitivity of tissue biopsy is low; it can be nondiagnostic in almost half of the cases. We propose that this patient's presentation, that of CNS vasculitis, represented the first clinical manifestation of an underlying MCTD or other connective tissue disease. Other findings consistent with MCTD or other connective tissue disease like systemic lupus erythematosus (SLE) may develop on careful followup. Intriguingly, Sato and coworkers reported that CSF anti-U1 RNP antibodies with an increased anti-U1 RNP index showed 64.3% sensitivity and 92.9% specificity for central neuropsychiatric SLE (NPSLE) [20]. 

 We also cannot rule out that some manifestations may have been present prior to this presentation due to the absence of a reliable history. Other viable possibilities include chronic vasculitis infection that could not be isolated especially with the suboptimal response to steroids. The course of the disease is too long for a paraneoplastic syndrome, but the possibility cannot be completely excluded. The presence of autoimmune biomarkers can be an incidental normal finding, but we suggest that the finding of persistently high titers of the anti-RNP antibody is less likely to be an incidental normal finding. Another plausible explanation is a late onset metabolic encephalopathy that could be related to familial genetic mutation or sporadic mutation. 

 The diagnosis in this case relies on the clinical picture, exclusion of other etiologies, the angiographic evidence, and the high titer of anti-RNP antibodies. We propose that theoretically, this case be added to the evidence supporting neurologic manifestations early in the course of MCTD similar to the case reported by Matsui and coinvestigators. MCTD be considered as a diagnosis in patients presenting with neurologic decline and/or suspected vasculitis of the CNS.

## 4. Conclusion

In this paper we report a case of chronic and progressive encephalopathy in a 49-year-old woman, in which after extensive evaluations, very high titers of anti-RNP antibodies were discovered, and biopsy results confirmed CNS vasculitis.

This case emphasizes the importance of CNS vasculitis as a diagnostic consideration in patients presenting with unexplained subacute to chronic, progressive neurologic symptoms. We propose further that this case illustrates the potential viability of MCTD as an underlying etiology of neurologic symptoms (despite the classic notion that MCTD tends to spare the CNS) even in the absence of clear systemic manifestations of connective tissue disease. Neurologists and other clinicians should be aware of the fact that neurological manifestations can be the earliest symptoms of many systemic diseases. Awareness of this fact may aid diagnosis early in the course of the disease, allowing initiation of appropriate treatments in a timely fashion to improve the likelihood of a good outcome.

##  Conflict of Interests

The authors declare that there are no financial disclosures or conflict of interests.
